# Transcriptome analysis reveals potential mechanisms underlying differential heart development in fast- and slow-growing broilers under heat stress

**DOI:** 10.1186/s12864-017-3675-9

**Published:** 2017-04-13

**Authors:** Jibin Zhang, Carl J Schmidt, Susan J Lamont

**Affiliations:** 1 Department of Animal Science, Iowa State University, 806 Stange Rd, 2255 Kildee Hall, Ames, IA 50011 USA; 2 Department of Animal and Food Sciences, University of Delaware, 531 South College Ave, Newark, DE 19716 USA

**Keywords:** RNA-seq, Broilers, Heart, Heat stress, Gene expression, Cell cycle, Illinois, Ross, Polo-like Kinase, Pathways

## Abstract

**Background:**

Modern fast-growing broilers are susceptible to heart failure under heat stress because their relatively small hearts cannot meet increased need of blood pumping. To improve the cardiac tolerance to heat stress in modern broilers through breeding, we need to find the important genes and pathways that contribute to imbalanced cardiac development and frequent occurrence of heat-related heart dysfunction. Two broiler lines – Ross 708 and Illinois – were included in this study as a fast-growing model and a slow-growing model respectively. Each broiler line was separated to two groups at 21 days posthatch. One group was subjected to heat stress treatment in the range of 35–37 °C for 8 h per day, and the other was kept in thermoneutral condition. Body and heart weights were measured at 42 days posthatch, and gene expression in left ventricles were compared between treatments and broiler lines through RNA-seq analysis.

**Results:**

Body weight and normalized heart weight were significantly reduced by heat stress only in Ross broilers. RNA-seq results of 44 genes were validated using Biomark assay. A total of 325 differentially expressed (DE) genes were detected between heat stress and thermoneutral in Ross 708 birds, but only 3 in Illinois broilers. Ingenuity pathway analysis (IPA) predicted dramatic changes in multiple cellular activities especially downregulation of cell cycle. Comparison between two lines showed that cell cycle activity is higher in Ross than Illinois in thermoneutral condition but is decreased under heat stress. Among the significant pathways (*P* < 0.01) listed for different comparisons, “Mitotic Roles of Polo-like Kinases” is always ranked first.

**Conclusions:**

The increased susceptibility of modern broilers to cardiac dysfunction under heat stress compared to slow-growing broilers could be due to diminished heart capacity related to reduction in relative heart size. The smaller relative heart size in Ross heat stress group than in Ross thermoneutral group is suggested by the transcriptome analysis to be caused by decreased cell cycle activity and increased apoptosis. The DE genes in RNA-seq analysis and significant pathways in IPA provides potential targets for breeding of heat-tolerant broilers with optimized heart function.

**Electronic supplementary material:**

The online version of this article (doi:10.1186/s12864-017-3675-9) contains supplementary material, which is available to authorized users.

## Background

When exposed to hot environment, livestock tend to promote heat loss through increasing blood flow toward skin. Therefore, changes in the cardiovascular system usually occurred to acclimate long-term thermal environment. However, such thermoregulatory response can only enable them survive within a limited range of thermal zone at the sacrifice of food production, despite the varied tolerance to heat stress between different animals [1]. Therefore, heat stress has caused billions of annual economic loss in livestock industry in the US [2] and is also becoming an imminent challenge to the world’s livestock industry due to the expected global warming.

Modern broilers with fast growth rate are susceptible to cardiac dysfunction due to small hearts relative to large body size. For example, the prevalence of arrhythmia in fast-growing broilers is 27%, but less than 1% in slow-growing broilers [3]. Although dietary restriction can reduce the occurrence of arrhythmia, ascites and sudden death syndrome, it also reduces the growth performance of these birds [3–5]. It would be advantageous to reduce the loss from heat stress in broiler industry, by optimizing cardiac development and body growth through genetic selection. To initiate the breeding of broilers resistant to heat stress and heart failure, it is important to identify significant genes and pivotal pathways responsible for the increased susceptibility to cardiac dysfunction and sensitivity to heat stress in fast-growing broilers compared to slow-growing broilers.

Schmidt et al. (2009) conducted a morphological comparison of body growth and tissue development between a modern broiler line Ross 708 and a legacy line called Illinois. The study showed that Ross broilers grow 1.8 and 1.3 times faster than Illinois broilers in body mass and heart mass respectively from 2 to 35 days posthatch. However, after 14 days posthatch, the percentage of heart mass relative to body mass became lower in Ross broilers than in Illinois broilers due to fast growth of body mass [6]. The Illinois line is a broiler line developed through crossing of progeny of New Hampshire males and females carrying the Columbia feather pattern [7–9]. After 1950s, Illinois broilers were maintained as closed population under relaxed selection. Therefore, it can serve as a slow-growing broiler model for comparison with modern fast-growing broilers to identify the genetic changes caused by intense selection.

The objective of this study is to characterize differences of gene expression in left ventricle of heart in response to heat stress through RNA-seq analysis in Ross broilers and Illinois broilers. We chose the left ventricle to study cardiac gene expression change in response to heat stress, because left ventricle failure has been suggested to play a significant primary role in the pathogenesis of ascites, which is a chronic heart-related disease occurring more frequently in modern fast-growing broilers than in slow-growing broilers [3]. Left ventricular failure could lead to ascites through increased pressure in pulmonary arteries [10]. Many fast-growing broilers show symptoms of left ventricular damage, such as degeneration of proteins related to cardiac muscle contraction, reduced fractional shortening and impaired ventricular wall motion [11]. Reduced size and pumping capacity has been observed in left ventricles but not right ventricles of preascitic broilers [12, 13]. In addition, the dilation of the left-sided heart has been observed in ascitic broilers due to increased hemodynamic burden [14]. When broilers are exposed to heat stress, their hearts would have higher pumping load and pressure to dissipate heat through increased blood circulation. Therefore, we investigated gene expression in the left ventricle of broilers in this study, expecting to see more change induced by heat stress and more differences between the two genetic lines in the left ventricle than in other parts of the heart. Through comparison of cardiac transcriptome response between the two lines, we aim to identify the important genes and pathways involved in cardiac functional regulation in fast-growing broilers that cause heat-related heart morbidity.

## Methods

### Broilers and heat stress treatment

Eggs of Ross 708 broilers from Mountaire farm in Millsboro, Delaware, and eggs of Illinois broilers from University of Illinois at Urbana-Champaign were hatched at the University of Delaware. After hatch, twenty male broilers from each line were randomly selected for this study to minimize gender specific effects, because male broilers are more susceptible to cardiac dysfunction than females [15]. The selected male broilers were initially maintained with *ad libitum* access to feed and drink in large colony houses warmed to 33 °C and the temperature was reduced by 3 °C each week until the house temperature reached 24 °C at 21 days posthatch. After 21 days posthatch, each broiler line was separated to two groups (*n* = 8-11 per group) with one group remain in the thermoneutral condition and the other group treated by heat stress (HS) in the range of 35–37 °C for 8 h/day. After euthanizing by cervical dislocation at 42 days of age, body and heart weights of the broilers were measured, and differences among groups were analyzed through two-way analysis of variance (ANOVA) and post hoc least significant difference (LSD) test in JMP Pro 12.0.1 (SAS Institute, Cary, NC). Finally, five to six broilers were selected from each group, and their left ventricles were collected, flash frozen in liquid nitrogen and stored at −80 °C for subsequent RNA isolation.

### Total RNA isolation

Total RNA samples were extracted from chicken left ventricular tissues using Qiagen RNeasy Fibrous Tissue Mini Kit following the manufacturer’s instruction. The concentration and overall quality of RNA samples were assessed by Nanodrop ND-100 spectrophotometer and Agilent 2100 Bioanalyzer, and only the samples with RNA Integrity Number above 9 were used for library construction.

### cDNA library construction and sequencing

A transcriptome library of each sample was constructed using Illumina TruSeq RNA Library Prep Kit (Illumina Inc., San Diego, CA) following the manufacturer’s instruction and sequenced by the HiSeq 2500 Sequencing System (Illumina) at the Delaware Biotechnology Institute’s Sequencing and Genotyping Center (Newark, DE). All libraries were sequenced at a depth of ~10 million, 50 bp single-end reads per library. The sequencing data have been deposited in NCBI’s Sequence Read Archive database with accession number SRP082125 (http://www.ncbi.nlm.nih.gov/sra/SRP082125).

### RNA-seq analysis

A series of applications in Discovery Environment of iPlant Collabrative (https://de.cyverse.org/DE), including FastQC (version 0.10.1), TopHat2-SE with TopHat (version 2.0.9) and Bowtie (version 2.1.0), and HTSeq-with-BAM-input with HTSeq (version 0.5.4) were utilized to for RNA-seq analysis. After quality assessment of the reads using FastQC, all libraries were of good quality with Phred score larger than 30 in nearly 100% of bases. Sequence reads in each library were mapped to *Gallus gallus* Galgal4.81 reference genome using TopHat2-SE with default parameters. The mapped reads per exon were then counted using HTSeq-with-BAM-input program with default parameters. The number of reads per gene was finally calculated and shown in the output file with Ensembl gene ID.

Principal component analysis (PCA) was performed using the Bioconductor package DEseq2 (version 1.10.1) in R software (version 3.1.3) based on variance-stabilized normalized read counts [16]. Differentially expressed (DE) genes between treatments and lines were obtained through analysis using edgeR (version 3.12.0). To minimize the effect of technical bias on the result, trimmed mean of M-values method was utilized to normalize numbers of reads in edge R [17]. A general linear model including treatment and line effects were fit to the data in edgeR. Log_2_ fold change (log_2_FC) and false discovery rate (FDR) determined by Benjamini-Hochberg method were calculated to filter significant DE genes. The genes with |log_2_FC| > 1 and FDR < 0.1 were defined as significant DE genes in a pair-wise comparison between different treatments or lines. To visualize the effect of heat stress, contrast (Ross_HS - Illinois_HS) - (Ross_Thermoneutral – Illinois_Thermoneutral) was used to compare the heat stress groups between the two lines. With the DE genes in each pair-wise comparison, changes in canonical pathways, regulation of cellular activities, and functions of organs were analyzed and predicted using Ingenuity pathway analysis (IPA) software (Ingenuity Systems, Redwood City, CA).

### Fluidigm Biomark assay

To validate RNA-seq results, Biomark assay was conducted with the same 23 RNA samples for 45 genes covering the whole range of log_2_ fold change in RNA-seq. These genes were selected based on their functional importance in literatures on cell cycle, cardiac function, cardiac development, cardiac inflammatory response, cardiomyocyte apoptosis, cardiac hypertrophy and cardiac heat stress response. The geometric mean of C_t_ values of three housekeeping genes [glyceraldehyde-3-phosphate dehydrogenase (*GAPDH*), hexose-6-phosphate dehydrogenase (*H6PD*) and ribosomal protein S13 (*RPS13*)] were used for normalization. Primers used for each gene are shown in Additional file 1: Table S1. Of each sample, 50 ng of RNA was used for cDNA preparation with Reverse Transcription Master Mix (Fluidigm, South San Francisco, CA) according to the manufacturer’s protocol. To determine the proper preamplification cycle number, 24 genes were randomly chosen to run on a Flex-Six integrated fluidic circuit (IFC) with cDNA pools of four groups separated by treatment and line. Each cDNA pool was preamplified for 10, 12 and 14 cycles, and 12 cycles was found to be the best preamplification cycle number that allows proper C_t_ value for most genes (data not shown). Then 48.48 IFC was used to run Biomark assay for individual samples in duplicate at 12 cycles. IFC was run in BioMark HD (Fluidigm) Real-Time PCR system and data was analyzed using Fluidigm Real-Time PCR Analysis software. One Gene [angiotensin II type-1 receptor (*AGTR1*)] with nonspecific amplification was identified by melting curve and excluded from analysis. C_t_ values of spots with no detection on the chip due to low expression were imputed as 30. Replicates showing shifted peak in melting curves were deleted. Gene expression comparison between different groups was done using 2 ^(−ΔΔCt)^ method in Excel and pairwise correlation and linear regression between Log_2_FC in Biomark assay and that in RNA-seq were calculated in JMP Pro 12.0.1 software.

## Results

### Change of body and heart weights of two broiler lines under heat stress

**Fig. 1:**
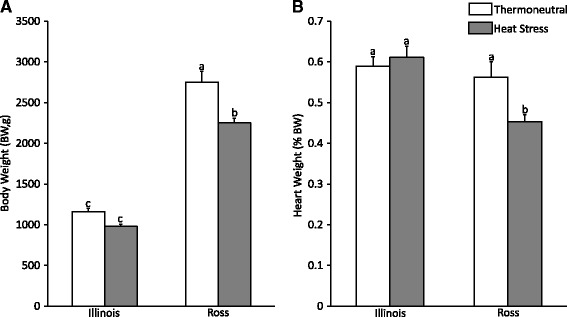
Body weight (**a**) and normalized heart weight (**b**) of two broiler lines under thermoneutral and heat stress conditions. Each bar represents Mean ± SEM. Different letters (a–c) indicate significant differences among different groups

In two-way ANOVA for comparison of body weight and relative heart mass, we found significant interaction between chicken line and treatment for both traits. This means that the effect of heat stress on body weight and relative heart weight always differ between lines. When we did a post-hoc LSD test, we found a significant decrease of body weight and relative heart weight induced by the 21-day cyclic heat stress only in Ross broilers (*P* < 0.05, Fig. 1). The body weight (BW) at 42 days posthatch was always more than 2 times higher in Ross broilers (*P* < 0.05) than in Illinois broilers regardless of whether they were under thermoneutral or heat stress condition (Fig. 1a). Unlike the difference in BW, there is no significant difference of normalized heart weight between the two broiler lines under thermoneutral condition. However, after exposure to heat stress for 21 days, Ross broilers showed a significant decrease in normalized heart weight (*P* < 0.05), while Illinois broilers did not change (Fig. 1b) compared to the thermoneutral group, indicating that the heart weight decreased faster than that of BW in Ross broilers under heat stress, but did not change significantly in Illinois broilers. Therefore, heat stress appears to have exerted a more deleterious effect on cardiac development in fast-growing than in slow-growing broilers.

### Validation of RNA-seq results with Biomark assay

**Fig. 2:**
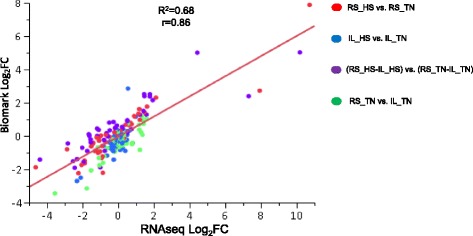
Linear regression fitted for Log_2_ Fold Change (FC) of selected genes determined via Biomark assay and RNA-seq technology. Different contrasts were marked in different colors. Different groups in the comparisons were labeled as Line_Treatment (RS: Ross, IL: Illinois, HS: heat stress, TN: thermoneutral). R-square and Pearson correlation coefficient are labeled as “R^2^” and “r”. Log_2_FC in Biomark assay equals -ΔΔC_t_ for each comparison. Average C_t_ value for each group was the means of samples in that group. Average expression of three housekeeping genes including *GAPDH*, *H6PD* and *RPS13* were used for normalization of C_t_ values

For the 44 test genes in 48.48 BioMark IFC, Pearson Product–moment Correlation between Biomark assay and RNA-seq Log_2_FCs is 0.86 (Fig. 2). Among the four comparisons between treatments and lines, Ross heat stress vs. thermoneutral comparison shows the highest correlation (0.92) between Biomark assay and RNA-seq Log_2_FCs, because most of the 44 genes showed differential expression in Ross heat stress group. As shown in Fig. 2, Log_2_FCs in Biomark assay agree very well with those in RNA-seq data no matter how small the log_2_FC is. The linear regression fitting the data accounts for more than 68% of total variation. However, for bone morphogenetic protein 10 (*BMP10*) and myosin heavy chain 7 (*MYH7*) which have the largest variation in expression among different groups, preamplification at 12 cycles can only render proper C_t_ value for heat stress group with high expression but no detection in some thermoneutral samples with low expression. With C_t_ values of some samples in thermoneutral group imputed as 30, Biomark assay Log_2_FCs would be much likely inaccurate and therefore pull the linear regression away from the ideal diagonal line (Fig. 2).

### Distinct response of cardiac gene expression to heat stress between the two broiler lines

Among the 10 million 50 bp single-end reads in each sample, an average of 94% reads were mapped to the reference genome, with 11% representing multiple mapping and 1.7% representing ambiguous assignment to multiple genomic features. On average, expression of 13,126 genes were detected in each individual, accounting for 77% of the 17,108 annotated genes in *Gallus gallus* Galgal4. 81 reference genome in the Ensembl database (Additional file 2: Table S2). Setting the threshold for read counts of each gene above 1 count per million in each sample of at least one treatment group, 11,279 genes were retained for differential expression analysis.

**Fig. 3:**
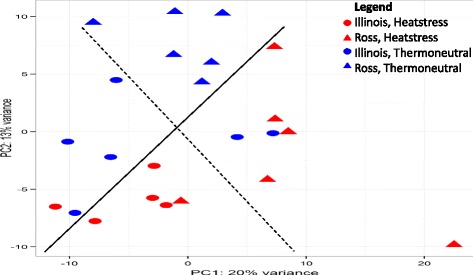
Principal component analysis (PCA) plot generated in DEseq2 showing variation within and between groups. Horizontal and vertical axis show two principal components that explain the greatest proportion (20 and 13%) of variation in variance-stabilized normalized counts. The solid and dash diagonal line respectively set a general division between treatments and lines respectively. Only Ross broilers in two treatment groups cluster separately. Different groups are represented in different shapes and colors: Ross (triangle), Illinois (circle), Heat stress (red), Thermoneutral (blue)

**Fig. 4:**
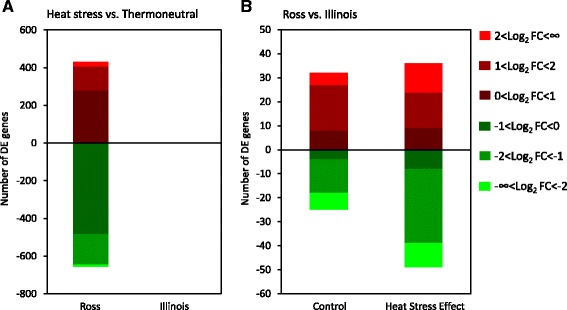
Number of significant differentially expressed (DE) genes with false discovery rate (FDR) < 0.1 in comparisons between two treatment groups within each line (**a**) and comparisons of inherent difference and heat stress effect between two broiler lines (**b**). Upregulated and downregulated DE genes are represented in red and blue color. DE genes within different range of Log_2_ fold change are represented in different brightness of the color

In the PCA plot, which visualizes similarity of samples with different groups, 33% of variation in read counts were explained by principal component 1 (PC1) and principal component 2 (PC2). Ross broilers were separated well by the diagonal line into two clusters based on treatment, but the separation of different treatment groups in Illinois broilers was not clear with two individuals in thermoneutral group and one individual in heat stress group intermingled (Fig. 3). This result also corresponds to the significant difference of DE gene number for heat stress vs. thermoneutral group between Ross and Illinois broilers. Setting FDR below 0.1, expressions of 1089 genes were significantly changed by heat stress treatment in Ross broilers when compared to the thermoneutral group (Fig. 4a). Among the 40% DE genes that were upregulated under heat stress, *BMP10* and *MYH7* showed highest log_2_FC around 10. Among the 60% downregulated DE genes under heat stress, myosin heavy chain 1E (*MYH1E*) and X Kell blood group precursor-related family, member 9 (*XKR9*) showed lowest log_2_FC around −4 (Additional file 3: Table S3). However, in Illinois broilers, heat stress treatment seems to have very little effect on gene expression. Only 4 genes showed significant change (Fig. 4a) with maximal log_2_FC around −2 in expression of tenascin C (*TNC*). Setting the threshold log_2_FC > 1.0, 152 upregulated DE genes and 173 downregulated DE genes were left in Ross broiler, but only 2 upregulated DE genes, prostaglandin D2 synthase (*PTGDS*) and WD repeat domain 83 opposite strand (*WDR830S*), and 1 downregulated DE gene, TNC, remained in Illinois broilers (Additional file 3: Table S3). This result indicates that the cardiac response of Ross broilers to heat stress is much more dramatic than that of Illinois broilers.

The cluster of two broiler lines within the same treatment group is also not very distinct, because two Illinois broilers in control group and one Ross broiler in heat stress group were intermixed with individuals of the other line (Fig. 3). As shown in Fig. 4b, there were only 67 significant DE genes between Ross and Illinois broilers under thermoneutral condition, with 32 genes upregulated and 35 genes downregulated. After normalization with deduction of difference between thermoneutral groups, 85 significant DE genes were identified between heat stress groups in the two lines, with 36 genes upregulated and 49 genes downregulated. Because the inherent differences between the two lines have been subtracted, the differential expression of these DE genes are solely caused by differential heat stress effect on the two lines. Among these DE genes, 45 and 68 genes show log_2_FC > 1 respectively under thermoneutral condition and heat stress treatment (Fig. 4b), and 14 genes were shared between the two groups of DE genes. Interestingly, all the 14 shared genes showed differential expressions of opposite directions under the two treatment conditions in the pairwise comparison between two lines (Table 1). Eight genes [stathmin 1 (*STMN1*), cell division cycle 45 (*CDC45*) and 20 (*CDC20*), DNA replication helicase/nuclease 2 (*DNA2*), cyclin B2 (*CCNB2*), deoxyadenosine kinase (*dAK*), mitotic kinesin-like protein 1 (*MKLP1*) and structural maintenance of chromosomes 2 (*SMC2*)] showed higher expression in Ross than in Illinois under thermoneutral condition, but their expressions were downregulated by heat stress effect in Ross broilers. As shown in Table 1, all these genes are required for cell cycle and division process, indicating that there is significant reduction in cell cycle activity in Ross broilers. Six genes [FXYD domain containing ion transport regulator 2 (*FXYD2*), beta-1-syntrophin (*SNTB1*), *KIAA1210*, avidin (*AVD*), *AGTR1* and P53 effector related to PMP-22 (*PERP*)] showed lower expression in Ross than in Illinois, but their expressions were upregulated by heat stress effect in Ross broilers. Among these genes, *FXYD2*, * SNTB1* and *AGTR1* are involved in regulation of cardiac function and morphology, *PERP* is a positive regulator of cell apoptosis, and functions of *AVD* and *KIAA1210 *are unknown (Table 1). Among the 14 genes, the gene with highest degree of expression change is *AGTR1*. Under thermoneutral condition, Ross broilers showed over 8 times lower expression of *AGTR1* in the left ventricle than Illinois broilers, but heat stress increased the expression of *AGTR1* more than 64 times in Ross compared to Illinois broilers (Table 1).

**Table 1:** Shared DE genes for Ross vs. Illinois between thermoneutral and heat stress conditions

Gene Symbol	Thermoneutral			Heat Stress			Gene Function
	*P*-value	Log_2_FC	FDR	*P*-value	Log_2_FC	FDR	
*STMN1*	5.30E-05	1.24	0.023	0.0003	−1.59	0.066	Regulates microtubule dynamics in cell division [105]
*CDC45*	5.11E-05	1.34	0.023	0.00046	−1.71	0.076	Key replication helicase cofactor in DNA replication [70]
*DNA2*	7.47E-06	1.71	0.0078	5.99E-05	−2.31	0.030	Essential DNA-helicase for chromosome replication [106]
*CCNB2*	0.00025	1.17	0.065	0.00042	−1.63	0.075	Important regulator required for cell mitosis [107]
*dAK*	0.00028	1.46	0.070	1.88E-08	−3.33	0.00011	Crucial regulator for DNA precursor synthesis [108]
*MKLP1*	6.14E-05	1.10	0.025	0.00046	−1.40	0.076	Mediates cell cycle cytokinesis [109]
*CDC20*	2.28E-06	1.58	0.0032	0.00029	−1.74	0.065	Regulates chromosome segregation and mitotic exit [110]
*SMC2*	0.00023	1.32	0.064	2.81E-06	−2.44	0.0042	Essential for chromosome segregation and condensation [111]
*FXYD2*	1.08E-08	−2.03	6.08E-05	3.36E-09	3.00	3.79E-05	Required for ion transport in heart [112]
*SNTB1*	7.48E-06	−1.09	0.0078	6.21E-05	1.41	0.030	Involved in muscle contraction [113]
*KIAA1210*	0.00017	−1.26	0.052	0.00011	1.87	0.046	Unknown
*AVD*	2.15E-06	−3.55	0.0032	2.05E-05	4.43	0.016	Unknown
*AGTR1*	0.00012	−3.51	0.041	2.95E-06	6.00	0.0042	Important role in pathogenesis of cardiac hypertrophy [85]
*PERP*	0.00012	−1.05	0.040	7.78E-06	1.78	0.0098	Mediates p53-dependent apoptosis [114]

*Log*_*2*_ *FC:* log_2_ fold change, *FDR:* False Discovery Rate

### Dramatic changes in multiple pathways in Ross broiler under heat stress

For canonical pathway analysis, IPA software takes into account all the DE genes with Log_2_FC > 1 and FDR < 0.1 in one comparison. Based on the number of DE genes and total mapped genes in one pathway, it calculates *p*-value through right-tailed Fisher Exact Test [18]. However, for the stacked bar chart shown in the result, IPA counts total number of both significant and nonsignificant DE genes. In this case, IPA takes into account totally 9,457 mapped DE gene out of 11,279 filtered genes. Eight significant (*p* < 0.01) pathways were listed for comparison Ross heat stress vs. Ross thermoneutral (Table 2). Interestingly, the top five among the eight pathways are all involved in cell cycle and mitosis (Fig. 4a). For example, “Mitotic Roles of Polo-like Kinase” regulates multiple processes from entry to exit of cell mitosis [19]; “Cell Cycle: G2/M DNA Damage Check Point Regulation” and “DNA Damage-induced 14-3-3σ Signaling” both regulate G2/M transition and ensure that no damage in DNA occurs during DNA replication before cell cycle proceeds to M phase [20, 21]; “ATM signaling” and “p53 signaling” are essential pathways for repair of DNA damage [22, 23], initiation of cell cycle arrest and cell apoptosis [24, 25]. Based on the match of observed regulation patterns of significant DE genes and the literature-derived regulation directions, IPA calculates an activation z-score to predict activation or inhibition of each pathway [26]. As shown in Table 2, “Mitotic Roles of Polo-like Kinase” pathway was predicted to be inhibited with negative z-score, while “Cell Cycle: G2/M DNA Damage Check Point Regulation” and “P53 signaling” pathways were predicted to be activated with positive z-score (Table 2). This result indicates that heat stress may have dramatic inhibiting effect on cell cycle activity in Ross broilers’ hearts.

**Table 2:**
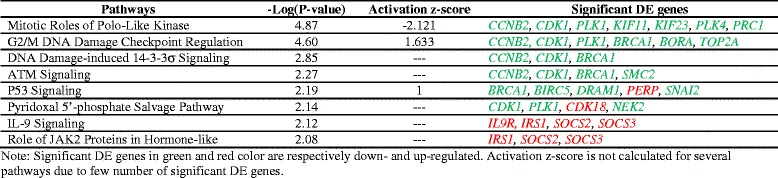
Significant pathways (*P* < 0.01) predicted from DE genes of Ross heat stress vs. Ross thermoneutral

In addition to inhibition of cell cycle activity, heat stress seems to have also caused disruption in immune system and cardiac functions of Ross broilers. Table 3 shows the top regulatory effects of DE genes for Ross heat stress vs. thermoneutral contrast on bio-functions and diseases that IPA predicted based on consistency of DE gene regulation and its literature database. Genes with large fold changes and great significance in each regulatory effect were shown as well. As shown in Table 3, in addition to inhibited cell proliferation and cell survival, IPA also predicted decreased leukocytes and inflammatory response from regulation of some genes related to immune function such as upregulation of suppressor of cytokine signaling 3 (*SOCS3*) and downregulation of T-cell surface glycoprotein CD4 (*CD4*), CD40 ligand (*CD40LG*) and signal transducer and activator of transcription 4 (*STAT4*). The high confidence of predicted disruption in immune system is consistent with the high ranks of immune-related pathways “IL-9 Signaling” and “Role of JAK2 in Hormone-like Cytokine Signaling” pathways in Table 2. Both pathways are ranked as significant because of the upregulation of insulin receptor substrate 1 (*IRS1*), suppressor of cytokine signaling 2 (*SOCS2*) and *SOCS3*. SOCS family has inhibitory effect on signaling transduction and negatively regulates IL-9 [27], JAK2 and IRS1 [28]. Therefore, upregulations of *SOCS2* and *SOCS3* may play an important role in inhibition of the immune system in Ross broilers under heat stress.

**Table 3:**
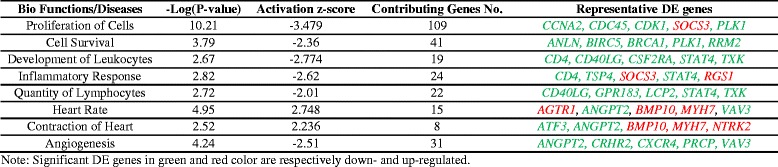
Predicted Effect (|z-score| > 2) of DE genes on biological functions and disease in Ross heat stress vs. Ross thermoneutral

Finally, IPA also predicted increased heart rate and cardiac contraction as well as decreased angiogenesis in Ross broilers’ left ventricle under heat stress compared to those under thermoneutral condition based on regulation of several genes related to cardiovascular function (Table 3). For example, *BMP10* [29] and *MYH7* [30] which are essential for contraction of cardiac muscle were upregulated, whereas angeopoietin-2 (*ANGPT2*) which decreases contractile function of cardiomyocytes [31] but increases angiogenesis in heart [32] was downregulated. The expression changes of these important regulators indicate possible pressure overload and disrupted angiogenesis which can eventually lead to cardiac hypertrophy and failure [33, 34].

### Different cell cycle activity in comparisons between two lines under different conditions

**Fig. 5:**
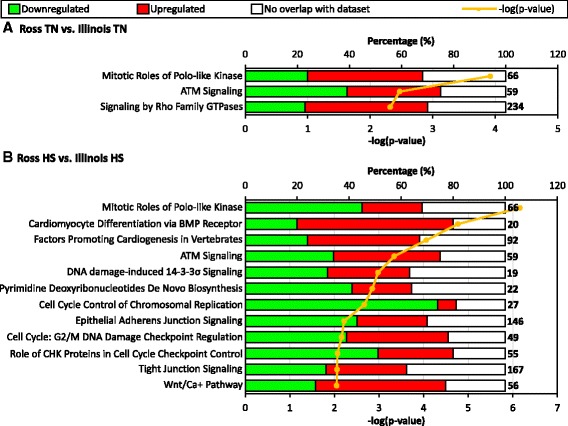
Top ranked canonical pathways (−log(p-value) > 2) in Ingenuity Pathway Analysis (IPA) for DE genes between two broiler lines in thermoneutral (TN) condition (**a**) and due to heat stress (HS) effect (**b**). –log (*p*-value) of each pathway is shown by orange plot. Both significant and nonsignificant DE genes were counted. Percentage of downregulated genes, upregulated genes and unannotated genes in each pathway are respectively represented in green, red and white color in each bar. Total number of genes in each pathway is shown at the end of each bar

With 67 significant DE genes (Log_2_FC > 1 and FDR < 0.1) between thermoneutral groups of the two broiler lines, IPA listed three significant canonical pathways (*P* < 0.01) (Fig. 5a). But for the 85 significant DE genes triggered by heat stress effect alone, twelve significant canonical pathways (*p* < 0.01) were shown in IPA (Fig. 5b), indicating that these genes have more diverse functions. However, a common interesting point is that the “Mitotic Roles of Polo-like Kinase” pathway is always ranked as the most significant pathway in both comparisons, the same as the comparison between heat stress and thermoneutral group in Ross broilers. In this pathway, the upregulated genes decreased from 43.9 to 22.7%, whereas the downregulated genes increased from 24.2 to 45.4% due to heat stress effect for comparison between Ross and Illinois (Fig. 5), indicating that the pathway might be inhibited by heat stress in Ross broilers.

**Fig. 6:**
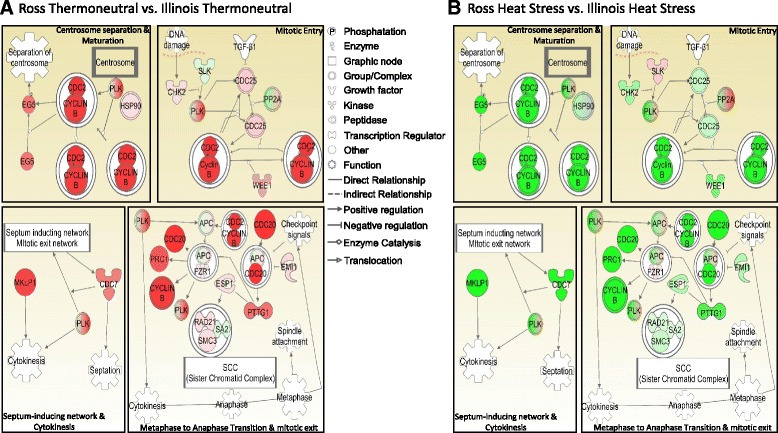
Different regulation of mitotic roles of polo-like kinase pathways in Ross vs. Illinois broilers under thermoneutral condition (**a**) and heat stress (**b**). Higher and lower expressions of genes in Ross than in Illinois broilers are shown respectively in red and green color. The complexes in both green and red contain multiple genes with higher or lower expression in Ross broilers. Unannotated genes in chicken are shown in white color. The intensity of the color indicates extent of differential expression. Different shapes of genes represent different molecular properties. Different types of arrows indicate different relationships

The polo-like kinases (PLKs) are an evolutionarily conserved family of enzymes involved in multiple phases of cell mitotic regulation (Fig. 5). At the entry of mitosis, PLKs can activate cell division cycle-25 (CDC25) through phosphorylation. The activated CDC25 can then dephosphorylate and activate the heterodimer formed by cell division cycle-2 (CDC2) and cyclin B which in turn induces further activation of CDC25, forming a positive feedback loop to amplify the activity of CDC25, CDC2 and cyclin B (Fig. 6). The amplified CDC25, CDC2 and cyclin B then promote the mitotic entry [19]. During maturation and separation of centrosome, PLKs are stabilized by heat shock protein-90 (HSP90) [35] and activated CDC2-cyclin B complex can phosphorylate and activate the plus-end-directed motor EG5 which promotes separation of chromosomes during mitosis [19]. In addition to separation of chromosomes, PLKs are also required for initiation of cytokinesis (Fig. 6). Through interaction with MKLP1, they can promote organization of the central spindle and formation of contractile ring [36]. Meanwhile, PLKs can induce septum formation and mitotic exit through kinase CDC7 [19]. At the transition from metaphase to anaphase, PLKs and CDC2-cyclin B complex can also activate anaphase-promoting complex (APC) which can induce proteasomal degradation of anaphase inhibitors [37, 38]. The complex formed by APC and fizzy/cell division cycle 20 related 1 (FZR1) can then trigger destruction of CDC20, cyclin B, PLKs and protein regulator of cytokinesis-1 (PRC1) [39–41]. PLK1 can activate APC through destruction of early mitotic inhibitor 1 (EMI1) [42]. The complex formed by APC and CDC20 can also cause degradation of pituitary tumor-transforming-1 (PTTG1) [43] which has inhibitory effect on the APC-FZR1 complex [44] and separin (ESP1) [45]. The released ESP1 from binding of PTTG1 can promote cleavage of sister chromatid complex (SCC) facilitating sister chromatid separation [19].

As shown in Fig. 6, under thermoneutral condition, Ross broilers showed higher expression than Illinois broilers in most of the key regulators in the pathway, such as MKLP1, CDC2, CDC20 and cyclin B. However, when the chickens were exposed to heat stress condition, the heat stress effect seems to have caused lower expression in most of the key regulators in Ross broilers than in Illinois broilers. Among these regulators, MKLP1, EG5, CDC2, CDC7, CDC20 and cyclin B showed great downregulation of expression in Ross broilers. Being affected by heat stress to such great extent, the mitotic pathways involved by PLKs may play an important role in regulation of cardiac response and contribute significantly to the decreased heart weight in Ross broilers under heat stress.

In addition to “Mitotic Roles of Polo-like Kinase” pathway, there are also some other cell cycle related pathways ranked as significant for Ross heat stress vs. Illinois heat stress (Fig. 6b), such as “ATM signaling”, “DNA damage-induced 14-3-3σ Signaling” and “Cell Cycle: G2/M DNA Damage Checkpoint Regulation” pathways which are also in top-rank pathways for Ross heat stress vs. Ross thermoneutral (Table 2). “ATM signaling” which is involved in the DNA-damage response, is also shared by Ross thermoneutral vs. Illinois thermoneutral comparison (Fig. 5a). From thermoneutral to heat stress condition, the upregulated genes increased 35.6–40.7%, whereas the downregulated genes decreased from 39.0% to 33.9% for Ross vs. Illinois contrast. Therefore, this pathway may be also involved in the retarded cardiac growth in Ross broilers under heat stress. Among the twelve significant pathways affected by heat stress for Ross vs. Illinois contrast, seven pathways are involved in cell cycle regulation, including the four aforementioned pathways and another three pathways regulating cell cycle progression - “Pyrimidine Deoxyribonucleotides De Novo Biosynthesis”, “Cell Cycle Control of Chromosomal Replication” and “Role of CHK Proteins in Cell Cycle Checkpoint Control” [46] pathways. Generally, for the pathways involved in the normal cell cycle progression, there are more genes with lower expression in Ross broilers than in Illinois broilers due to heat stress effect. Such pattern is especially obvious for “Cell Cycle Control of Chromosomal Replication” pathway, for which there are 74.1% downregulated genes but only 18.5% upregulated genes with annotation (Fig. 5b). This result further indicates the inhibited cell cycle in hearts of Ross broilers under heat stress.

## Discussion

The greater reduction of growth rate in commercial broilers than the slow-growing broilers under heat stress reported in the present study agree well with the previous reports [47, 48], further indicating higher susceptibility of modern broilers than low BW broilers to heat stress. The undermined growth performance under heat stress is certainly related to various changes in different tissues, such as reported intestinal injury and decreased relative weight of bursa, thymus and spleen [49]. In the current study, we also observed reduced relative weight of heart in commercial broilers under heat stress and identified some correlate DE genes and altered pathways. To the best of our knowledge, this is the first study investigating cardiac response to heat stress in broilers through transcriptome analysis. With 21-day cyclic heat stress treatment, we have been able to see how differently the fast-growing broilers (Ross) and slow-growing broilers (Illinois) adapt to thermal stress conditions. In Illinois broilers, heat stress only induced three significant DE genes compared to thermoneutral group, so these birds may have already adapted to the heat stress and they no longer need to alter gene expression to adjust their physiological function. Among the three DE gene, *WDR830S* is a novel gene with unknown function. *TNC* is an essential promoter of cardiac angiogenesis [50] and its expression can be induced by mechanical overload [51]. Expression of *PTGDS* can be induced in vascular endothelial cells by laminar fluid shear stress and thus is associated with progression of atherosclerosis [52]. Because these genes only showed significant change in Illinois broilers but mild change in Ross broilers in the heat stress vs. thermoneutral comparison (Additional file 3: Table S3), the upregulation of *TNC* and downregulation of *PTGDS* in Illinois broilers may be important for their better adaption to heat stress than Ross broilers.

In the fast-growing broilers, more than 300 DE genes were identified, with most genes related to cell cycle regulation. According to prediction of IPA, several pathways in cell cycle promotion, such as “Mitotic Roles of Polo-like Kinase”, were downregulated, and several pathways in cell cycle arrest, such as “p53 signaling” in cell cycle, were upregulated. Therefore, it is highly possible that the slowed cardiac growth in Ross broilers is due to inhibited cell proliferation and increased cell apoptosis. Hyperthermia-induced apoptosis has been long-recognized in various cell lines and wildly utilized in cancer treatment [53–55]. Previous studies suggested that hyperthermia-induced apoptosis is characterized by the occurrence of internucleosomal DNA damage [56]. It occurred during G_1_ phase of cell cycle in rat MFH-2NR cells and mouse FM3A cells [57, 58], and both G1 phase and S phase in human HL-60 cells [59, 60]. As the heat load increased with time or temperature, the apoptosis apparently transitions to necrosis, leading to pathological cell damage and an inflammatory response in the tissue [60–62]. However, different cell types have varied resistance to hyperthermia-induced apoptosis [56]. For example, in normal rats treated with whole-body hyperthermia at 41.5 °C for 2 h, the highest apoptosis occurred in thymus, but only negligible apoptosis occurred in heart, lung and liver [63]. The different resistance to hyperthermia-induced apoptosis in different tissues may be related to different regulation mechanisms.

Previous study suggested that commercial broilers exposed to daily 7-h cycles of 35 °C showed physiological response to reduce hyperthermia-induced apoptosis in liver with four genes showing anti-apoptosis regulation among 40 DE genes [64]. But the Ross broilers exposed to daily 8-h cycles of 35–37 °C in this study remarkably showed apoptosis in heart with upregulation of *PERP* and sphingomyelin phosphodiesterase 3 (*SMPD3*), and downregulation of *CDC7*, *CDC45*, thymidylate synthase (*TYMS*), *STMN1*, ribonucleotide reductase M2 (*RRM2*), urokinase-plasminogen activator (*PLAU*), TRAF interacting protein (*TRAIP*), snail family zinc finger 2 (*SNAI2*) and NDC80 kinetochore complex component SPC25 (*SPC25*) (Additional file 3: Table S3). PERP is an important mediator of P53-dependent apoptotic pathway and it is directly activated by P53 and highly expressed in cells undergoing apoptosis [65]. SMPD3 catalyzes hydrolysis from sphingomyelin to ceramide [66] which accumulates in response to stress stimuli such as heat stress and promotes cell cycle arrest and apoptosis [67]. CDC7 is an essential cell cycle regulator, and its inactivation can lead to S-phase arrest and P53-dependent apoptosis in mouse embryonic stem cell culture [68]. Being rescued by an ectopically expressed transgene, CDC7 (−/−) mice could survive but showed decreased cell proliferation, impaired organ development and reduced body size [69]. CDC45 is a key replication helicase cofactor required for DNA replication, and its depletion was suggested to slow S-G2 progression in HCT116 cells [70]. TYMS and RRM2 are both indispensable enzymes for DNA synthesis. TYMS catalyzes the production of dTMP from dUMP [71], while RRM2 is the regulatory subunit of ribonucleotide reductase which catalyzes the conversion from ribonucleotides to deoxyribonucleotides [72]. Because both dTMP and deoxyribonucleotides are necessary for DNA replication and repair, inhibition of TYMS and RRM2 has been suggested to induce apoptosis in cancer cells [73, 74]. STMN1 is a key regulator in interphase and late mitosis to prevent assembly and promote disassembly of microtubule. Its upregulation has been observed in various cancers and its inhibition has been proposed as a therapeutic way for cancer treatment [75–77]. PLAU regulates cell growth and apoptosis through regulation of several growth factors and control of cell-matrix contacts. Depletion of *PLAU* reduces cell proliferation and increases cell death in cell culture and during tissue regeneration [78]. TRAIP can inhibit cell death through inhibition of mediation of nuclear factor kappa-B by tumor necrosis factor receptor associated factor 2 [79]. TRAIP-deficient mice exhibited impaired embryonic development due to proliferative defects and excessive apoptosis [80]. SNAI2 functions as a survival factor that can prevent apoptosis of damaged cells. Disruption of *SNAI2* can sensitize the tumor cells to apoptosis signaling and delay the development of mammary gland [81, 82]. As a component of NDC80 kinetochore complex, SPC25 is required not only to establish and maintain kinetochore-microtubule attachment in mitotic spindle and metaphase alignment of chromosomes, but also to move chromosomes to spindle poles in anaphase [83]. Depletion of *SPC25* can cause aberrant spindles, aberrant mitosis and increased apoptosis in human HeLa cells [84]. Most of these genes are mainly involved in cell cycle progression from S phase to M phase and regulated by P53 signaling. Together with the highest ranks of “Mitotic Roles of Polo-like Kinase” and “G2/M DNA Damage Checkpoint” pathways, we speculate that the hyperthermia-induced apoptosis in the heart of Ross broilers may be P53-dependent and occur primarily in S-M phase. To confirm this speculation, in vivo cellular studies are still necessary in the future.

Due to the relatively smaller heart caused by cell cycle arrest and apoptosis, there also seems to be regulation of adaptive hypertrophy to cope with hypertension in the adverse situation of slow cardiac growth. The large increase of *AGTR1* expression in Ross broilers under heat stress is an indicator of this change (Table 1), because overexpression of *AGTR1* in cardiomyocytes has been reported to induce cardiac hypertrophy and remodeling [85]. In addition, downregulation of vav guanine nucleotide exchange factor 3 (*VAV3*) may also indicate cardiac dysfunction in Ross broilers under heat stress, because *VAV3*-deficient mice exhibited left ventricular hypertrophy, systemic arterial hypertension and tachycaridia [86]. BMP10, a cardiac cytokine expressed restrictively in the developing and postnatal heart, is essential for regulation of cardiac growth and chamber maturation [29]. Previous study suggested that BMP10 provides a positive growth signal for cardiomyocytes antagonizing cell cycle inhibitors, and regulates several key cardiogenic factors to maintain adequate cardiac function [29]. In the current study, *BMP10* showed the most significant upregulation (more than 2,000 times higher) in the heart of Ross broilers under heat stress compared to the thermoneutral group. Upregulation of *BMP10* has been reported in mice with myocardial hypertrophy and excessive trabeculation [87]. Therefore, BMP10 in Ross broilers may provide compensatory regulation through counteracting upregulated cell cycle inhibitors to promote cardiac hypertrophy under heat stress. Another indicator of possible cardiac remodeling is expression change of genes encoding different myosin heavy chain isoforms (MHCs). *MYH7*, which encodes β-MHC in slow fibers of cardiac ventricles [88], was upregulated more than 300 times in heart of Ross broilers under heat stress. Whereas *MYH1E*, which is an ortholog of human MYH4 and encodes MHC-IIb in fast fibers in skeletal muscle, was downregulated about 25 times by heat stress in Ross broilers. In human suffering from chronic heart failure, decrease of MHC-I encoded by MYH7 but increase of MHC-IIb was detected in costal diaphragm [89]. Therefore, the upregulation of *MYH7* in Ross broilers’ hearts under heat stress in this study may also indicate a propensity to heart failure in these birds, because decrease in the ratio of fast MHC and slow MHC in cardiac muscle can reduce myofibrillar Ca2 + −activated ATPase activity and systolic function of heart [90]. The increase of slow MHC and decrease of fast MHC under continuous mild heat stress has also been reported in myoblast cell culture of human, mouse [91] and quail [92]. Therefore, the pro-slow shifting of MHC isoforms may be common response to heat stress in muscle cells both in vitro and in vivo.

Another concern in fast-growing broilers under heat stress is the possible inhibitory effect on immune system. Decreased leukocytes and antibody production in peripheral blood has been reported in 31-week-old laying chickens under heat stress at 35 °C for 5 weeks [93]. In Ross broilers, heat stress in the range of 35-37 °C from 35 to 42 days posthatch also decreased T-helper (CD4^+^), T-cytotoxic (CD8^+^) lymphocytes and antibody titer in peripheral blood [94]. Therefore, similar inhibition effects of heat stress on the immune system may also exist in cardiac tissue, which is indicated by the expression changes of several key regulators of immune cell development, such as downregulation of *CD4*, *CD40LG*, lymphocyte cytosolic protein 2 (*LCP2*), *STAT4*, thrombospondin 4 (*TSP4*) and G protein-coupled receptor 183 (*GPR183*) and upregulation of *SOCS3*. Regarding their functions, we found the downregulated genes are generally important for cardiac immune response, while SOCS3 is an inhibitor of inflammatory response. CD4 plays a major role in cardiac allograft rejection, so anti-CD4 therapy is usually needed for a successful heart allograft [95]. Anti-CD40LG antibody reduced myocardial inflammation response in mice with acute viral myocarditis [96]. Deficiency of *LCP2* has been found to lead to malfunction of mast cells and mild tachycardia in mouse heart [97]. *STAT4*-deficient mice are resistant to induction of myocarditis by cardiac myosin immunization [98]. TSP4 plays an important role in regulation of vascular inflammation. Knockout of *TSP4* led to a reduced number of macrophages in aortic root lesions and reduced inflammatory factors in vascular walls [99]. GPR183 is highly regulated during cardiac inflammation, and may play key roles in pathogenesis of cardiovascular disease, inflammation and autoimmune diseases [100]. Antagonists of GPR183 have been suggested to be tested in paradigms relevant for cardiovascular diseases [101]. On the other hand, SOCS3 attenuated proinflammatory signaling mediated by activator of STAT family proteins, playing a negative role in a variety of inflammatory and autoimmune processes [102]. Therefore, the expression changes of all these genes point to decreased inflammatory response induced by heat stress in the heart of Ross broilers as predicted by IPA (Table 3).

Another merit of this study is the inclusion of a heritage line – Illinois. Through comparison between two broiler lines, we can determine how diversity in genetics and growth trait can lead to differential response to heat stress. When the two broiler lines are under thermoneutral condition, Ross broilers showed faster growth of BW with similar normalized heart weight compared to Illinois broilers, so their cardiac development must be faster than Illinois broilers to keep pace with their fast body growth. When the two broiler lines were under heat stress, normalized heart weight showed little change in Illinois but significant reduction in Ross broilers, indicating the slowed cardiac development of Ross under heat stress. However, to compare the heat stress effect on the two broiler lines, the intrinsic difference between thermoneutral groups must be subtracted for normalization. Through this approach, we identified “Mitotic Roles of Polo-like Kinase” Pathway as the primary pathway that contribute to the differential growth rate between the two broiler lines pre and post heat stress treatment. Expression of polo-like kinase has been reported to be highly correlated with proliferative activity of cardiomyocytes in rat [103]. Therefore, the lower expression of *PLK1* and *PLK4* (Table 2) in Ross broilers under heat stress compared to those under thermoneutral condition may be also an indicator of decreased proliferation of cardiomyocytes. PLK1 plays an important role in recovery of cells from G2 DNA damage-induced arrest in mammals. Overexpression of *PLK1* enables cells to override cell cycle checkpoint even the DNA damage is still present [104]. Therefore, PLK1 may be a key regulator of cellular adaption to heat-induced DNA damage, and the downregulated polo-like kinases in Ross broilers under heat stress may be a key mechanism of the increased cell-cycle arrest and cell apoptosis.

## Conclusions

This is the first study with extensive investigation of gene expression in heart affected by heat stress and genetic composition using RNA-seq technology. Significant decrease of normalized heart weight and dramatic change of gene expression were found in Ross broilers but not Illinois broilers. Decreased cell cycle activity, increased cell apoptosis, disruption of heart rate and contraction, impaired immune cell development and retarded angiogenesis were predicted to be induced by heat stress in Ross broilers’ hearts based on enrichment analysis of DE genes. “Mitotic Roles of Polo-like Kinase” was identified as the most significant pathway that is responsible for change of cell cycle activity and cell apoptosis induced by heat stress. Regulation of S to M phase in cell cycle seems to be affected the most by heat stress in Ross broilers’ hearts based on enrichment of the significant pathways. Finally, switch of fast/slow MHC balance was also speculated from functions of DE genes and pathways. Additional gene expression studies at protein level and more immunohistochemistry studies at cellular level are still needed in the future to testify these hypotheses. The results of this study narrows the targets for future studies on genetic regulation of cardiac function under heat stress. The identified important genes including *BMP10*, *MYH7* and *PLK1* and the "Mitotic Roles of Polo-like Kinases" pathway could be prospective markers and targets for selection and breeding of heat-tolerant broilers.

## Additional files

Additional file 1: Table S1. Primers used in Biomark 48.48 IFC for the validations of RNA-seq data (PDF 110 kb)

Additional file 2: Table S2. Statistical summary of sequence reading, mapping and counting (PDF 103 kb)

Additional file 3: Table S3. Significant DE genes (FDR < 0.1, |log_2_FC| < 1) from EdgeR for comparisons between heat stress and thermoneutral groups in Ross 708 and Illinois broilers (XLSX 38 kb)

## Abbreviations

AGTR1: Angiotensin II type-1 receptor
APC: Anaphase-promoting complex
BW: Body weight
CCNB2: Cyclin B2
CD40LG: CD40 ligand
CDC: Cell division cycle
DE: Differentially expressed
FDR: False discovery rate
HS: Heat stress
IFC: Integrated fluidic circuit
IPA: Ingenuity Pathway Analysis
log_2_FC: Log_2_ fold change
MKLP1: Mitotic Kinesin-Like Protein 1
MYH1E: Myosin heavy chain 1E
PCA: Principal component analysis
PERP: P53 effector related to PMP-22
PLK: Polo-like kinase
PTGDS: Prostaglandin D2 synthase
RNA-seq: RNA sequencing
SMC2: Structural maintenance of chromosomes 2
SOCS: Suppressor of cytokine signaling
STMN1: Stathmin 1
TNC: tenascin C

## References

[1] Renaudeau D, Collin A, Yahav S, de Basilio V, Gourdine JL, Collier RJ (2012). Adaptation to hot climate and strategies to alleviate heat stress in livestock production. Animal.

[2] St-Pierre NR, Cobanov B, Schnitkey G (2003). Economic losses from heat stress by US livestock industries. J Dairy Sci.

[3] Olkowski AA (2007). Pathophysiology of heart failure in broiler chickens: structural, biochemical, and molecular characteristics. Poult Sci.

[4] Baghbanzadeh A, Decuypere E (2008). Ascites syndrome in broilers: physiological and nutritional perspectives. Avian Pathol.

[5] Sahraei M (2014). Effects of feed restriction on metabolic disorders in broiler chickens: a review. Res J Biol Sci.

[6] Schmidt CJ, Persia ME, Feierstein E, Kingham B, Saylor WW (2009). Comparison of a modern broiler line and a heritage line unselected since the 1950s. Poult Sci.

[7] Schoettle CE, Reber EF, Norton HW, Alberts JO (1956). A study of new hampshire x barred columbian chicks from 2 day of age to 10 weeks of age. 2. Effect of coccidiostats at 10 weeks of age. Poult Sci.

[8] Schoettle CE, Reber EF, Alberts JO, Scott HM (1956). A study of new hampshire x barred columbian chicks from 2 days of age to 10 weeks of age. 1. Growth - organ weight - liver fat and protein - femur and tibia fat and ash of chicks fed a ration free of antibiotics and coccidiostats. Poult Sci.

[9] Waterhouse HN, Scott HM (1962). Effect of sex, feathering, rate of growth and acetates on chicks need for glycine. Poult Sci.

[10] Wideman RF, Rhoads DD, Erf GF, Anthony NB (2013). Pulmonary arterial hypertension (ascites syndrome) in broilers: A review. Poult Sci.

[11] Olkowski AA, Duke T, Wojnarowicz C (2005). The aetiology of hypoxaemia in chickens selected for rapid growth. Comp Biochem Physiol A Mol Integr Physiol.

[12] Martinez-Lemus LA, Miller MW, Jeffrey JS, Odom TW (1998). Echocardiographic evaluation of cardiac structure and function in broiler and leghorn chickens. Poult Sci.

[13] Olkowski AA, Korver D, Rathgeber B, Classen HL (1999). Cardiac index, oxygen delivery, and tissue oxygen extraction in slow and fast growing chickens, and in chickens with heart failure and ascites: a comparative study. Avian Pathol.

[14] Wu DJ, Lin JA, Chiu YT, Cheng CC, Shyu CL, Ueng KC, Huang CY (2003). Pathological and biochemical analysis of dilated cardiomyopathy of broiler chickens - an animal model. Chin J Physiol.

[15] Olkowski AA, Classen HL (1998). High incidence of cardiac arrhythmias in broiler chickens. Zentralbl Veterinarmed A.

[16] Anders S, Huber W (2010). Differential expression analysis for sequence count data. Genome Biol.

[17] Robinson MD, Oshlack A (2010). A scaling normalization method for differential expression analysis of RNA-seq data. Genome Biol.

[18] Jia P, Kao CF, Kuo PH, Zhao Z (2011). A comprehensive network and pathway analysis of candidate genes in major depressive disorder. BMC Syst Biol.

[19] Donaldson MM, Tavares AAM, Hagan IM, Nigg EA, Glover DM (2001). The mitotic roles of Polo-like kinase. J Cell Sci.

[20] Loebrich M, Jeggo PA (2007). The impact of a negligent G2/M checkpoint on genomic instability and cancer induction. Nat Rev Cancer.

[21] Morrison DK (2009). The 14-3-3 proteins: integrators of diverse signaling cues that impact cell fate and cancer development. Trends Cell Biol.

[22] Goodarzi AA, Noon AT, Deckbar D, Ziv Y, Shiloh Y, Loebrich M, Jeggo PA (2008). ATM signaling facilitates repair of DNA double-strand breaks associated with heterochromatin. Mol Cell.

[23] Rappold I, Iwabuchi K, Date T, Chen JJ (2001). Tumor suppressor p53 binding protein 1 (53BP1) is involved in DNA damage-signaling pathways. J Cell Biol.

[24] Harris SL, Levine AJ (2005). The p53 pathway: positive and negative feedback loops. Oncogene.

[25] Zhang YG, Ma WY, Kaji A, Bode AM, Dong ZG (2002). Requirement of ATM in UVA-induced signaling and apoptosis. J Biol Chem.

[26] Kraemer A, Green J, Pollard J, Tugendreich S (2014). Causal analysis approaches in Ingenuity Pathway Analysis. Bioinformatics.

[27] Lejeune D, Demoulin JB, Renauld JC (2001). Interleukin 9 induces expression of three cytokine signal inhibitors: cytokine-inducible SH2-containing protein, suppressor of cytokine signalling (SOCS)- 2 and SOCS-3, but only SOCS-3 overexpression suppresses interleukin 9 signalling. Biochem J.

[28] Ilangumaran S, Ramanathan S, Rottapel R (2004). Regulation of the immune system by SOCS family adaptor proteins. Semin Immunol.

[29] Chen HY, Shi S, Acosta L, Li WM, Lu J, Bao SD, Chen ZA, Yang ZC, Schneider MD, Chien KR, Conway SJ, Yoder MC, Haneline LS, Franco D, Shou W (2004). BMP10 is essential for maintaining cardiac growth during murine cardiogenesis. Development.

[30] Lankford EB, Epstein ND, Fananapazir L, Sweeney HL (1995). Abnormal contractile properties of muscle-fibers expressing beta-myosin heavy-chain gene-mutations in patients with hypertrophic cardiomyopathy. J Clin Invest.

[31] Greulich S, Maxhera B, Vandenplas G, de Wiza DH, Smiris K, Mueller H, Heinrichs J, Blumensatt M, Cuvelier C, Akhyari P, Ruige JB, Ouwens DM, Eckel J (2012). Secretory products from epicardial adipose tissue of patients with type 2 diabetes mellitus induce cardiomyocyte dysfunction. Circulation.

[32] Munk VC, de Miguel LS, Petrimpol M, Butz N, Banfi A, Eriksson U, Hein L, Humar R, Battegay EJ (2007). Angiotensin II induces angiogenesis in the hypoxic adult mouse heart in vitro through an AT(2)-B-2 receptor pathway. Hypertension.

[33] Gomez AM, Valdivia HH, Cheng H, Lederer MR, Santana LF, Cannell MB, McCune SA, Altschuld RA, Lederer WJ (1997). Defective excitation-contraction coupling in experimental cardiac hypertrophy and heart failure. Science.

[34] Shiojima I, Sato K, Izumiya Y, Schiekofer S, Ito M, Liao RL, Colucci WS, Walsh K (2005). Disruption of coordinated cardiac hypertrophy and angiogenesis contributes to the transition to heart failure. J Clin Invest.

[35] Blank M, Mandel M, Keisari Y, Meruelo D, Lavie G (2003). Enhanced ubiquitinylation of heat shock protein 90 as a potential mechanism for mitotic cell death in cancer cells induced with hypericin. Cancer Res.

[36] Liu XQ, Zhou T, Kuriyama R, Erikson RL (2004). Molecular interactions of Polo-like-kinase 1 with the mitotic kinesin-like protein CHO1/MKLP-1. J Cell Sci.

[37] Wang Q, Xie S, Chen J, Fukasawa K, Naik U, Traganos F, Darzynkiewicz Z, Jhanwar-Uniyal M, Dai W (2002). Cell cycle arrest and apoptosis induced by human polo-like kinase 3 is mediated through perturbation of microtubule integrity. Mol Cell Biol.

[38] Golan A, Yudkovsky Y, Hershko A (2002). The cyclin-ubiquitin ligase activity of cyclosome/APC is jointly activated by protein kinases Cdk1-cyclin B and Plk. J Biol Chem.

[39] Hauf S, Roitinger E, Koch B, Dittrich CM, Mechtler K, Peters JM (2005). Dissociation of cohesin from chromosome arms and loss of arm cohesion during early mitosis depends on phosphorylation of SA2. PLoS Biol.

[40] Archambault V, Glover DM (2009). Polo-like kinases: conservation and divergence in their functions and regulation. Nat Rev Mol Cell Biol.

[41] Reddy SK, Rape M, Margansky WA, Kirschner MW (2007). Ubiquitination by the anaphase-promoting complex drives spindle checkpoint inactivation. Nature.

[42] Hansen DV, Loktev AV, Ban KH, Jackson PK (2004). Plk1 regulates activation of the anaphase promoting complex by phosphorylating and triggering SCF beta TrCP-dependent destruction of the APC inhibitor Emi1. Mol Biol Cell.

[43] K-i I, Watanabe Y (2007). Chromosome cohesion in mitosis and meiosis. J Cell Sci.

[44] Hilioti Z, Chung YS, Mochizuki Y, Hardy CFJ, Cohen-Fix O (2001). The anaphase inhibitor Pds1 binds to the APC/C-associated protein Cdc20 in a destruction box-dependent manner. Curr Biol.

[45] Waizenegger IC, Gimenez-Abian JF, Wernic D, Peters JM (2002). Regulation of human separase by securin binding and autocleavage. Curr Biol.

[46] Pietenpol JA, Stewart ZA (2002). Cell cycle checkpoint signaling: Cell cycle arrest versus apoptosis. Toxicology.

[47] Cooper MA, Washburn KW (1998). The relationships of body temperature to weight gain, feed consumption, and feed utilization in broilers under heat stress. Poult Sci.

[48] Berrong SL, Washburn KW (1998). Effects of genetic variation on total plasma protein, body weight gains, and body temperature responses to heat stress. Poult Sci.

[49] Quinteiro-Filho WM, Ribeiro A, Ferraz-de-Paula V, Pinheiro ML, Sakai M, Sa LRM, Ferreira AJP, Palermo-Neto J (2010). Heat stress impairs performance parameters, induces intestinal injury, and decreases macrophage activity in broiler chickens. Poult Sci.

[50] Ballard VLT, Sharma A, Duignan I, Holm JM, Chin A, Choi R, Hajjar KA, Wong SC, Edelberg JM (2006). Vascular tenascin-C regulates cardiac endothelial phenotype and neovascularization. FASEB J.

[51] Yamamoto K, Dang QN, Kennedy SP, Osathanondh R, Kelly RA, Lee RT (1999). Induction of tenascin-C in cardiac myocytes by mechanical deformation - Role of reactive oxygen species. J Biol Chem.

[52] Taba Y, Sasaguri T, Miyagi M, Abumiya T, Miwa Y, Ikeda T, Mitsumata M (2000). Fluid shear stress induces lipocalin-type prostaglandin D-2 synthase expression in vascular endothelial cells. Circ Res.

[53] Arai Y, Kondo T, Tanabe K, Zhao QL, Li FJ, Ogawa R, Li M, Kasuya M (2002). Enhancement of hyperthermia-induced apoptosis by local anesthetics on human histiocytic lymphoma U937 cells. J Biol Chem.

[54] Katschinski DM, Robins HI, Schad M, Frede S, Fandrey J (1999). Role of tumor necrosis factor alpha in hyperthermia-induced apoptosis of human leukemia cells. Cancer Res.

[55] Overgaard J (1989). The current and potential role of hyperthermia in radiotherapy. Int J Radiat Oncol Biol Phys.

[56] Honma T (1996). Characteristics of hyperthermia-induced apoptotic cell death. Nippon Rinsho Jpn J Clin Med.

[57] Yonezawa M, Otsuka T, Matsui N, Tsuji H, Kato KH, Moriyama A, Kato T (1996). Hyperthermia induces apoptosis in malignant fibrous histiocytoma cells in vitro. Int J Cancer.

[58] Zhu WG, Aramaki R, Cai Y, Antoku S (1996). Promotion of heat-induced apoptosis in FM3A cells by protease inhibitors. Biochem Biophys Res Commun.

[59] Takasu T, Lyons JC, Park HJ, Song CW (1998). Apoptosis and perturbation of cell cycle progression in an acidic environment after hyperthermia. Cancer Res.

[60] Lim CU, Zhang Y, Fox MH (2006). Cell cycle dependent apoptosis and cell cycle blocks induced by hyperthermia in HL-60 cells. Int J Hyperthermia.

[61] Hildebrandt B, Wust P, Ahlers O, Dieing A, Sreenivasa G, Kerner T, Felix R, Riess H (2002). The cellular and molecular basis of hyperthermia. Crit Rev Oncol Hematol.

[62] Harmon BV, Corder AM, Collins RJ, Gobe GC, Allen J, Allan DJ, Kerr JFR (1990). Cell-death induced in a murine mastocytoma by 42–47° C heating in vitro - evidence that the form of death changes from apoptosis to necrosis above a critical heat load. Int J Radiat Biol.

[63] Sakaguchi Y, Stephens LC, Makino M, Kaneko T, Strebel FR, Danhauser LL, Jenkins GN, Bull JMC (1995). Apoptosis in tumors and normal-tissues induced by whole-body hyperthermia in rats. Cancer Res.

[64] Coble DJ, Fleming D, Persia ME, Ashwell CM, Rothschild MF, Schmidt CJ, Lamont SJ (2014). RNA-seq analysis of broiler liver transcriptome reveals novel responses to high ambient temperature. BMC Genomics.

[65] Attardi LD, Reczek EE, Cosmas C, Demicco EG, McCurrach ME, Lowe SW, Jacks T (2000). PERP, an apoptosis-associated target of p53, is a novel member of the PMP-22/gas3 family. Genes Dev.

[66] Krut O, Wiegmann K, Kashkar H, Yazdanpanah B, Kronke M (2006). Novel tumor necrosis factor-responsive mammalian neutral sphingomyelinase-3 is a C-tail-anchored protein. J Biol Chem.

[67] Hannun YA, Luberto C (2000). Ceramide in the eukaryotic stress response. Trends Cell Biol.

[68] Kim JM, Nakao K, Nakamura K, Saito I, Katsuki M, Arai K, Masai H (2002). Inactivation of Cdc7 kinase in mouse ES cells results in S-phase arrest and p53-dependent cell death. EMBO J.

[69] Kim JM, Takemoto N, Arai K, Masai H (2003). Hypomorphic mutation in an essential cell-cycle kinase causes growth retardation and impaired spermatogenesis. EMBO J.

[70] Rodriguez R, Gagou ME, Meuth M (2008). Apoptosis induced by replication inhibitors in Chk1-depleted cells is dependent upon the helicase cofactor Cdc45. Cell Death Differ.

[71] Berger FG, Berger SH (2006). Thymidylate synthase as a chemotherapeutic drug target - Where are we after fifty years?. Cancer Biol Ther.

[72] D’Angiolella V, Donato V, Forrester FM, Jeong YT, Pellacani C, Kudo Y, Saraf A, Florens L, Washburn MP, Pagano M (2012). Cyclin F-mediated degradation of ribonucleotide reductase M2 controls genome integrity and DNA repair. Cell.

[73] Backus HHJ, Wouters D, Ferreira CG, van Houten VMM, Brakenhoff RH, Pinedo HM, Peters GJ (2003). Thymidylate synthase inhibition triggers apoptosis via caspases-8 and-9 in both wild-type and mutant p53 colon cancer cell lines. Eur J Cancer.

[74] Rahman MA, Amin RMR, Wang D, Koenig L, Nannapaneni S, Chen Z, Wang Z, Sica G, Deng X, Chen ZG, Shin DM (2013). RRM2 regulates Bcl-2 in head and neck and lung cancers: a potential target for cancer therapy. Clin Cancer Res.

[75] Li J, Hu GH, Kong FJ, Wu KM, He B, Song K, Sun WJ (2014). Reduced STMN1 expression induced by RNA interference inhibits the bioactivity of pancreatic cancer cell line Panc-1. Neoplasma.

[76] Wang S, Akhtar J, Wang Z (2015). Anti-STMN1 therapy improves sensitivity to antimicrotubule drugs in esophageal squamous cell carcinoma. Tumour Biol.

[77] Zhu HW, Jiang D, Xie ZY, Zhou MH, Sun DY, Zhao YG (2015). Effects of stathmin 1 silencing by siRNA on sensitivity of esophageal cancer cells Eca-109 to paclitaxel. Genet Mol Res.

[78] Alfano D, Franco P, Vocca I, Gambi N, Pisa V, Mancini A, Caputi M, Carriero MV, Iaccarino I, Stoppelli MP (2005). The urokinase plasminogen activator and its receptor - Role in cell growth and apoptosis. Thromb Haemost.

[79] Lee SY, Choi Y (1997). TRAF-interacting protein (TRIP): A novel component of the tumor necrosis factor receptor (TNFR)- and CD30-TRAF signaling complexes that inhibits TRAF2-mediated NF-kappa B activation. J Exp Med.

[80] Park ES, Choi S, Kim JM, Jeong Y, Choe J, Park CS, Choi Y, Rho J (2007). Early embryonic lethality caused by targeted disruption of the TRAF-interacting protein (TRIP) gene. Biochem Biophys Res Commun.

[81] Wu WS, Heinrichs S, Xu D, Garrison SP, Zambetti GP, Adams JM, Look AT (2005). Slug antagonizes p53-mediated apoptosis of hematopoietic progenitors by repressing puma. Cell.

[82] Nassour M, Idoux-Gillet Y, Selmi A, Come C, Faraldo MLM, Deugnier MA, Savagner P (2012). Slug controls stem/progenitor cell growth dynamics during mammary gland morphogenesis. PLoS One.

[83] McCleland ML, Kallio MJ, Barrett-Wilt GA, Kestner CA, Shabanowitz J, Hunt DF, Gorbsky GJ, Stukenberg PT (2004). The vertebrate Ndc80 complex contains Spc24 and Spc25 homologs, which are required to establish and maintain kinetochore-microtubule attachment. Curr Biol.

[84] Bharadwaj R, Qi W, Yu HT (2004). Identification of two novel components of the human NDC80 kinetochore complex. J Biol Chem.

[85] Paradis P, Dali-Youcef N, Paradis FW, Thibault G, Nemer M (2000). Overexpression of angiotensin II type I receptor in cardiomyocytes induces cardiac hypertrophy and remodeling. Proc Natl Acad Sci U S A.

[86] Sauzeau V, Sevilla MA, Rivas-Elena JV, de Alava E, Montero MJ, Lopez-Novoa JM, Bustelo XR (2006). Vav3 proto-oncogene deficiency leads to sympathetic hyperactivity and cardiovascular dysfunction. Nat Med.

[87] Nakano N, Hori H, Abe M, Shibata H, Arimura T, Sasaoka T, Sawabe M, Chida K, Arai T, Nakahara K, Kubo T, Sugimoto K, Katsuya T, Ogihara T, Doi Y, Izumi T, Kimura A (2007). Interaction of BMP10 with Tcap may modulate the course of hypertensive cardiac hypertrophy. Am J Physiol Heart Circ Physiol.

[88] Walsh R, Rutland C, Thomas R, Loughna S (2010). Cardiomyopathy: a systematic review of disease-causing mutations in myosin heavy chain 7 and their phenotypic manifestations. Cardiology.

[89] Tikunov B, Levine S, Mancini D (1997). Chronic congestive heart failure elicits adaptations of endurance exercise in diaphragmatic muscle. Circulation.

[90] Tardiff JC, Hewett TE, Factor SM, Vikstrom KL, Robbins J, Leinwand LA (2000). Expression of the beta (slow)-isoform of MHC in the adult mouse heart causes dominant-negative functional effects. Am J Physiol Heart Circ Physiol.

[91] Yamaguchi T, Suzuki T, Arai H, Tanabe S, Atomi Y (2010). Continuous mild heat stress induces differentiation of mammalian myoblasts, shifting fiber type from fast to slow. Am J Physiol Cell Physiol.

[92] Choi YM, Chen PR, Shin S, Zhang J, Hwang S, Lee K (2016). Mild heat stress enhances differentiation and proliferation of Japanese quail myoblasts and enhances slow muscle fiber characteristics. Poult Sci.

[93] Mashaly MM, Hendricks GL, Kalama MA, Gehad AE, Abbas AO, Patterson PH (2004). Effect of heat stress on production parameters and immune responses of commercial laying hens. Poult Sci.

[94] Khajavi M, Rahimi S, Hassan ZM, Kamali MA, Mousavi T (2003). Effect of feed restriction early in life on humoral and cellular immunity of two commercial broiler strains under heat stress conditions. Br Poult Sci.

[95] Raisanen-Sokolowski A, Glysing-Jensen T, Mottram PL, Russell ME (1997). Sustained anti-CD4/CD8 treatment blocks inflammatory activation and intimal thickening in mouse heart allografts. Arterioscler Thromb Vasc Biol.

[96] Seko Y, Takahashi N, Azuma M, Yagita H, Okumura K, Yazaki Y (1998). Expression of costimulatory molecule CD40 in murine heart with acute myocarditis and reduction of inflammation by treatment with anti-CD40L/B7-1 monoclonal antibodies. Circ Res.

[97] Pivniouk VI, Martin TR, Lu-Kuo JM, Katz HR, Oettgen HC, Geha RS (1999). SLP-76 deficiency impairs signaling via the high-affinity IgE receptor in mast cells. J Clin Invest.

[98] Afanasyeva M, Wang Y, Kaya Z, Stafford EA, Dohmen KM, Sadighi Akha AA, Rose NR (2001). Interleukin-12 receptor/STAT4 signaling is required for the development of autoimmune myocarditis in mice by an interferon-gamma-independent pathway. Circulation.

[99] Frolova EG, Pluskota E, Krukovets I, Burke T, Drumm C, Smith JD, Blech L, Febbraio M, Bornstein P, Plow EF, Stenina OI (2010). Thrombospondin-4 regulates vascular inflammation and atherogenesis. Circ Res.

[100] Sun SQ, Liu CL (2015). 7 alpha, 25-dihydroxycholesterolmediated activation of EBI2 in immune regulation and diseases. Front Pharmacol.

[101] Gessier F, Preuss I, Yin H, Rosenkilde MM, Laurent S, Endres R, Chen YA, Marsilje TH, Seuwen K, Nguyen DG, Sailer AW (2014). Identification and characterization of small molecule modulators of the Epstein-Barr virus-induced gene 2 (EBI2) receptor. J Med Chem.

[102] Jo D, Liu DY, Yao S, Collins RD, Hawiger J (2005). Intracellular protein therapy with SOCS3 inhibits inflammation and apoptosis. Nat Med.

[103] Georgescu SP, Komuro I, Hiroi Y, Mizuno T, Kudoh S, Yamazaki T, Yazaki Y (1997). Downregulation of polo-like kinase correlates with loss of proliferative ability of cardiac myocytes. J Mol Cell Cardiol.

[104] van Vugt M, Bras A, Medema RH (2004). Polo-like kinase-1 controls recovery from a G2 DNA damage-induced arrest in mammalian cells. Mol Cell.

[105] Rubin CI, Atweh GF (2004). The role of stathmin in the regulation of the cell cycle. J Cell Biochem.

[106] Budd ME, Choe WC, Campbell JL (1995). DNA2 encodes a DNA helicase essential for replication of eurkaryotic chromosomes. J Biol Chem.

[107] Brandeis M, Rosewell I, Carrington M, Crompton T, Jacobs MA, Kirk J, Gannon J, Hunt T (1998). Cyclin B2-null mice develop normally and are fertile whereas cyclin B1-null mice die in utero. Proc Natl Acad Sci U S A.

[108] Eriksson S, Munch-Petersen B, Johansson K, Eklund H (2002). Structure and function of cellular deoxyribonucleoside kinases. Cell Mol Life Sci.

[109] Kuriyama R, Gustus C, Terada Y, Uetake Y, Matuliene J (2002). CHO1, a mammalian kinesin-like protein, interacts with F-actin and is involved in the terminal phase of cytokinesis. J Cell Biol.

[110] Sivakumar S, Gorbsky GJ (2015). Spatiotemporal regulation of the anaphase-promoting complex in mitosis. Nat Rev Mol Cell Biol.

[111] Strunnikov AV, Hogan E, Koshland D (1995). SMC2, a Saccharomyces cerevisiae gene essential for chromosome segregation and condensation, defines a subgroup within the SMC family. Genes Dev.

[112] Therien AG, Pu HX, Karlish SJ, Blostein R (2001). Molecular and functional studies of the gamma subunit of the sodium pump. J Bioenerg Biomembr.

[113] Ahn AH, Yoshida M, Anderson MS, Feener CA, Selig S, Hagiwara Y, Ozawa E, Kunkel LM (1994). Cloning of human basic A1, a distinct 59-kDa dystrophin-associated protein encoded on chromosome 8q23-24. Proc Natl Acad Sci U S A.

[114] Ihrie RA, Reczek E, Horner JS, Khachatrian L, Sage J, Jacks T, Attardi LD (2003). Perp is a mediator of p53-dependent apoptosis in diverse cell types. Curr Biol.

