# Supplementary material for: Transcriptome analysis reveals potential mechanisms underlying differential heart development in fast- and slow-growing broilers under heat stress
Source: BMC Genomics. 2017 Apr 13;18:295. doi: 10.1186/s12864-017-3675-9 (PMC5390434; doi:10.1186/s12864-017-3675-9)
Supplement: Supplementary file 1 — Primers used in Biomark 48.48 IFC for the validations of RNA-seq data (PDF 110 kb) [file 12864_2017_3675_MOESM1_ESM.pdf]

Primers used in Biomark 48.48 IFC for the validations of RNA-seq data

Gene	Gene Name	Forward Primer	Reverse Primer
AGTR1	Angiotensin II Receptor Type 1	TTTGCCTGAGCAAGAAGGACTA	TGTGGCCTTGTTTCAGGGAA
ANGPT2	Angiopoietin 2	CTGAGCAGACCCGCAAATTA	AGGGAATGTTCCAAAAGCTGAA
AVD	Avidin	TTCACAGGCACCTACATCACA	GGTGTTTTGTGTCCCATGCA
BAG3	BCL2 Associated Athanogene 3	ACCACAACAGCCGAACCA	GATGGGCCATTTGCTGATGAC
BMP10	Bone Morphogenetic Protein 10	GGACATCTATGCCATCTGCAAA	CGAACTCCTATGACGCCAAC
BRCA1	Breast Cancer 1, Early Onset	TGCTGCTGTGGACCTTTCA	TTCACTACAGAGGCACCACAAA
CASP3	Caspase 3, Apoptosis-Related Cysteine Peptidase	CGCTCAGGGGAAGATGTATCA	CCAGAGTCCACAGACTTGCTA
CASP8	Caspase 8, Apoptosis-Related Cysteine Peptidase	GCTTGTAACAGAGGGGCAAA	CACAGATGATGCCAGCCAAA
CCNB2	Cyclin B2	CTCCAGCTGCTGTGAATCAA	ACATCCATAGGGACAGGAGAC
CD4	T-Cell Surface Glycoprotein CD4	AGTGGAACCTGGATGTGTCA	TTTCCAAGCGTTCCTTCTCAAA
CD40LG	CD40 Ligand	CCAGTGAGGAGTTGCCAAAA	GCCACAGATGTCTCATTCCTAC
CDC20	Cell Division Cycle 20	TCAGCTGGAACAGCTACATCC	GCTCAGCCACTCTGACATCA
CDK1	Cyclin-Dependent Kinase 1	CCACAGCCATGGAGGATTACA	TGTGGCGCCCTTTATACACA
CDKN1A	Cyclin-Dependent Kinase Inhibitor 1A	TCTCCCCAGCGCTTGAAA	CTTGGGCTTATCGTGGACAAC
CXCR4	Chemokine (C-X-C motif) receptor 4	TATGGACGGCAGCATGGA	AATCTCCTCCGAGCCATTGT
FAS	Fas Cell Surface Death Receptor	GGTGTGAACATTGCGAGTCA	GCACACAGTGTCTGAAGTTGAA
FOXM1	Forkhead Box M1	GTGAAGCAAGGCATGGAGAA	GGTATGGAAACAGCAGCAGAA
FST	Follistatin	AGCCCGAACTTGAAGTCCAATA	GAGCTGCCTGGGCATAAAAC
GAPDH	Glyceraldehyde-3-Phosphate Dehydrogenase	GTGCTGGCATTGCACTGAA	CACAACACGGTTGCTGTATCC
GATA4	GATA Binding Protein 4	AAACAGAACCAGGGCTCTCA	TCCAGACATGGCAGAGACC
GATA6	GATA Binding Protein 6	TCAGGTCAAGATGGGCTGTAC	ACCAGTGATCCTGCCTGAC
H6PD	Hexose-6-Phosphate Dehydrogenase	ATGTACCGGGTGGACCACTA	AACTGACGGTTCTGATCTCGAAA
HSF2	Heat Shock Transcription Factor 2	AGGACTTCCAGGCCATGTTA	CATCTGCACGGAGCTTGTAA
HSP90AA1	Heat Shock Protein 90kDa Alpha Class A Member 1	ACACATGCCAACCGCATTTA	CCTCCTCAGCAGCAGTATCA
HSPA5	Heat Shock 70kDa Protein 5	TTTCTGCCATGGTCCTGACAA	AGGCTGGCACAGTAACAACA
HSPA8	Heat Shock 70kDa Protein 8	CTGGCAAGGAGAACAAGATCAC	TTCCTGAACCATCCGCTCAA
LCP2	Lymphocyte Cytosolic Protein 2	TCCTCTACCAGGCAACAATGAC	GGGGTTTCGTGCTTCTGTCTA
MYBL1	Myb-Like Protein 1	ATGGAACACTTGCACACTCA	TCTGGTGAGGCATACTGGTA
MYH7	Myosin Heavy Chain 7	ATTTCTGCCCAGCTCCAGAA	TGCCTCTAGTTCCTCCTCCA
MYH1E	Myosin Heavy Chain 1E	GATTCCCCAGCAGAGTCCTA	ATTGTCCCTCTGGGATAGCA
NES	Nestin	AGCAGCTCATGCACCTCA	GGGTGCTCTCTGCTTCCA
NTRK2	Neurotrophic Tyrosine Kinase Receptor Type 2	CACTTCTCCGGATGTTTCCAA	ATACCAGTGCTGCAATTCCC
PERP	P53 Apoptosis Effector Related To PMP22	CGCAGTCATCGCATTCTCAA	TTTCACTGGGTAGATGACCAAC
PLK1	Polo-Like Kinase 1	AGCCAAACCCTCTGAGAGAA	TGCTAACCCAGAAGATGGGAA
RPS13	Ribosomal Protein S13	CTTGCTAAGAAAGGCTTGACTCC	CAAAACGAACCTGGGCAACA
RRM2	Ribonucleotide Reductase M2	TCTCTGGAAGGCAAGACCAA	GTTGTCTGTGGGCTTTGACA
SIK1	Salt Inducible Kinase 1	GAGCAGCAGAGAGGGAAGAAA	CCTCCTCAGCAGCAGTATCA
SMAD6	SMAD Family Member 6	GTGTGCTGCAATCCGTACC	TTAGGAGACAGCCGGGAGTA
SMC2	Structural Maintenance Of Chromosomes 2	GAAGAGCTCGACCGAAAGAA	GCATCGAGAAGATGGAACCA
SNTB1	Syntrophin Beta 1	GTCAGAGACCAGCTGGGTAA	CAGTGCTTCTCGTTGTCTCC
SOCS2	Suppressor Of Cytokine Signaling 2	CGTGCTGATGTGCAAGGAC	ACGTGTACAGGGGTTTGTTCA
TBX5	T-Box 5	GTGGGGACGGAGATGATCATAA	GGATTGAGTCCAGTGACCTTCA
TGFB2	Transforming Growth Factor, Beta 2	CGTGCTCTAGATGCTGCCTA	GCCAAGATCCCTCTTGAAGTCA

TGFB3	Transforming Growth Factor, Beta 3	GGGCCCTGGATACCAACTAC	GGTCCTGTCGGAAGTCAATGTA
TLR4	Toll-Like Receptor 4	CCTGCTGGCAGGATGCA	TGTTCTGTCCTGTGCATCTGAA
TNC	Tenascin C	CAAAGGGCAACCAAGCAACA	TGCTGTCACTCCAATGCCATA
TNNT3	Troponin T Type 3	GCAAGCCCTTGAACATTGAC	ACCAGTCCCACAGTTCCTTA
VAV3	Vav 3 Guanine Nucleotide Exchange Factor	ACGTCCTGCAAAGTCTGTCA	CCAGCTCCACACTTAGAGCATA
