# Supplementary material for: Transcriptome analysis reveals potential mechanisms underlying differential heart development in fast- and slow-growing broilers under heat stress
Source: BMC Genomics. 2017 Apr 13;18:295. doi: 10.1186/s12864-017-3675-9 (PMC5390434; doi:10.1186/s12864-017-3675-9)
Supplement: Supplementary file 2 — Statistical summary of sequence reading, mapping and counting (PDF 103 kb) [file 12864_2017_3675_MOESM2_ESM.pdf]

Statistical summary of sequence reading, mapping and counting

Lines	Treatment	Bird No.	Raw Reads	Mapped Reads	% of Mapped Reads	Detected Genes	Transcriptome
Illinois	Heat Stress	1	10,000,000	9,475,810	94.8%	13,155	76.89%
Illinois	Heat Stress	2	10,000,000	9,397,562	94.0%	13,031	76.17%
Illinois	Heat Stress	3	10,000,000	9,495,394	95.0%	13,216	77.25%
Illinois	Heat Stress	4	10,000,000	9,358,232	93.6%	13,089	76.51%
Illinois	Heat Stress	5	10,000,000	9,462,325	94.6%	12,952	75.71%
Illinois	Thermoneutral	1	10,000,000	9,586,139	95.9%	12,914	75.49%
Illinois	Thermoneutral	2	10,000,000	9,412,525	94.1%	13,220	77.27%
Illinois	Thermoneutral	3	10,000,000	9,485,005	94.9%	13,001	75.99%
Illinois	Thermoneutral	4	10,000,000	9,441,373	94.4%	13,337	77.96%
Illinois	Thermoneutral	5	10,000,000	9,489,277	94.9%	13,107	76.61%
Illinois	Thermoneutral	6	10,000,000	9,506,032	95.1%	13,212	77.23%
Ross	Heat Stress	1	10,000,000	9,309,495	93.1%	13,218	77.26%
Ross	Heat Stress	2	10,000,000	9,432,475	94.3%	13,190	77.1%
Ross	Heat Stress	3	10,000,000	9,340,377	93.4%	13,270	77.57%
Ross	Heat Stress	4	10,000,000	9,284,131	92.8%	13,033	76.18%
Ross	Heat Stress	5	10,000,000	9,347,441	93.5%	13,503	78.93%
Ross	Heat Stress	6	10,000,000	9,329,626	93.3%	13,155	76.89%
Ross	Thermoneutral	1	10,000,000	9,329,547	93.3%	13,099	76.57%
Ross	Thermoneutral	2	10,000,000	9,433,942	94.3%	13,074	76.42%
Ross	Thermoneutral	3	10,000,000	9,364,674	93.6%	12,656	73.98%
Ross	Thermoneutral	4	10,000,000	9,394,578	93.9%	13,104	76.6%
Ross	Thermoneutral	5	10,000,000	9,396,177	94.0%	13,038	76.21%
Ross	Thermoneutral	6	10,000,000	9,467,707	94.7%	13,327	77.9%
